# On-X mitral valve leaflet embolization presenting as acute pulmonary oedema

**DOI:** 10.1186/s43044-022-00270-0

**Published:** 2022-05-04

**Authors:** Lindiwe Sidali, Nandipha Ndibi, Manogran Moopanar, Rajhmun Madansein

**Affiliations:** Department of Cardiothoracic Surgery, Inkosi Albert Luthuli Central Hospital, Nelson R Mandela School of Medicine, University of Kwa Zulu Natal, Durban, South Africa

**Keywords:** On-X, Mitral, Mechanical valve

## Abstract

**Background:**

Structural valve failure in the form of leaflet fracture and embolization is an uncommon complication. Severe valve regurgitation with pulmonary oedema in a patient with a history of a prosthetic cardiac valve requires urgent diagnosis. An echocardiogram is essential in identifying the cause of valve failure.

**Case presentation:**

We report a case of a 21-year-old male patient who presented with acute pulmonary oedema due to fracture and embolization of a leaflet of an On-X mitral valve four years post-implant. He underwent an emergency redo mitral valve replacement with a good outcome.

**Conclusions:**

Structural valve failure with leaflet fracture and embolization is an extremely rare complication and may be fatal. Good clinical acumen is required for prompt diagnosis in any patient who presents with a circulatory collapse in the presence of a history of previous cardiac surgery. Once the diagnosis is made, the patient should undergo emergency redo valve replacement surgery.

## Background

The most common complications of mechanical valves such as thromboembolism, prosthetic valve endocarditis, pannus formation and bleeding due to the use of anticoagulants are commonly described [1–6]. Structural valve failure in the form of leaflet fracture and embolization is an extremely rare complication and may be potentially fatal [2–4]. The cases of leaflet fracture and embolization reported in the literature occurred with the Bjork–Shiley tilting disc prosthetic heart valve, Edwards-Duromedics prosthesis and TRI Technologies mechanical valve, which are no longer on the market [1–5]. Leaflet embolization with an On-X mechanical valve (Cryolife, Kennesaw, GA) is uncommon, with only three cases described in the literature [3, 4, 6]. We describe the fourth case four years post-implant.

## Case presentation

A 21-year-old male patient presented to a local hospital with acute respiratory distress due to pulmonary oedema. He had a previous history of mitral valve replacement with a 25/33 mm On-X valve four years prior for mixed mitral valve disease due to rheumatic heart disease. A month prior to presentation, his international normalized ratio (INR) was subtherapeutic at 1.54, and his warfarin was adjusted. He presented with sudden onset of severe shortness of breath, dyspnoea grade IV and minor haemoptysis. On arrival at a local hospital, he had signs of severe respiratory distress with a saturation of 75% on room air and hypotension. He required resuscitation: intubation, ventilation, intravenous diuretics, and inotropes (dobutamine).

**Fig. 1:**
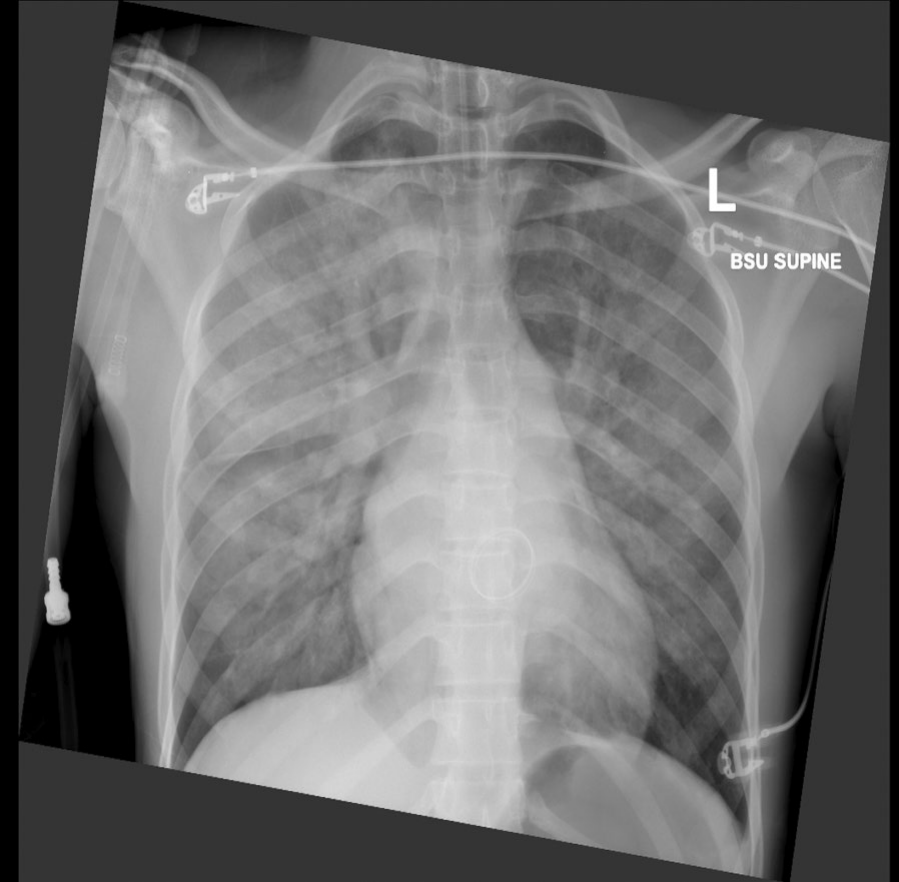
Chest radiograph showing pulmonary oedema

Once stabilized for transport, he was transferred by air to our centre (300kms) with suspected mechanical valve dysfunction. The laboratory findings were unremarkable except for an INR of 1.73. His chest radiograph showed fulminant pulmonary oedema (Fig. 1).

**Fig. 2:**
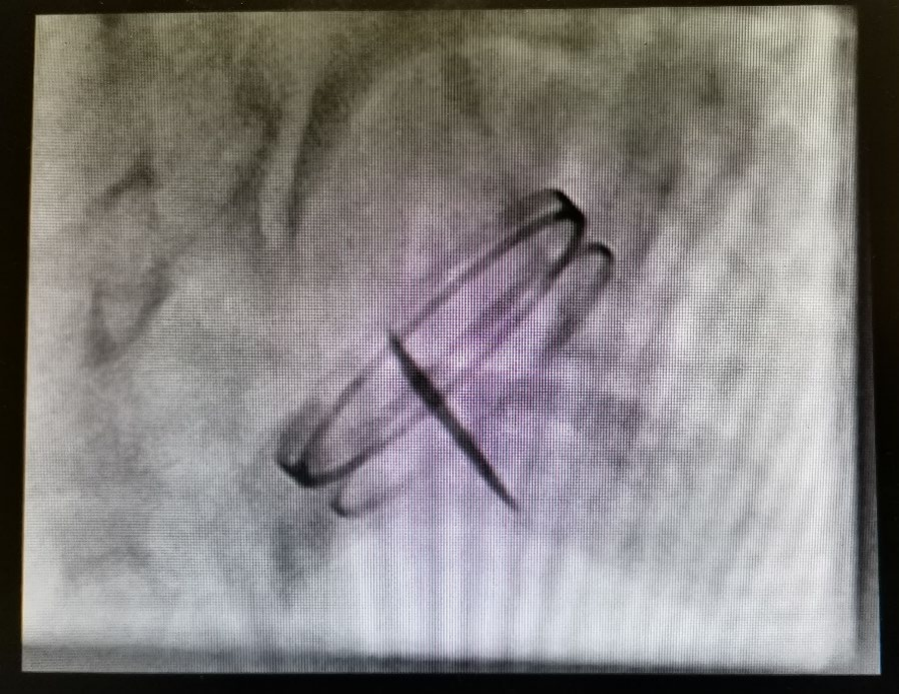
Fluoroscopy valve screen showing a single leaflet of the mechanical mitral valve

**Fig. 3:**
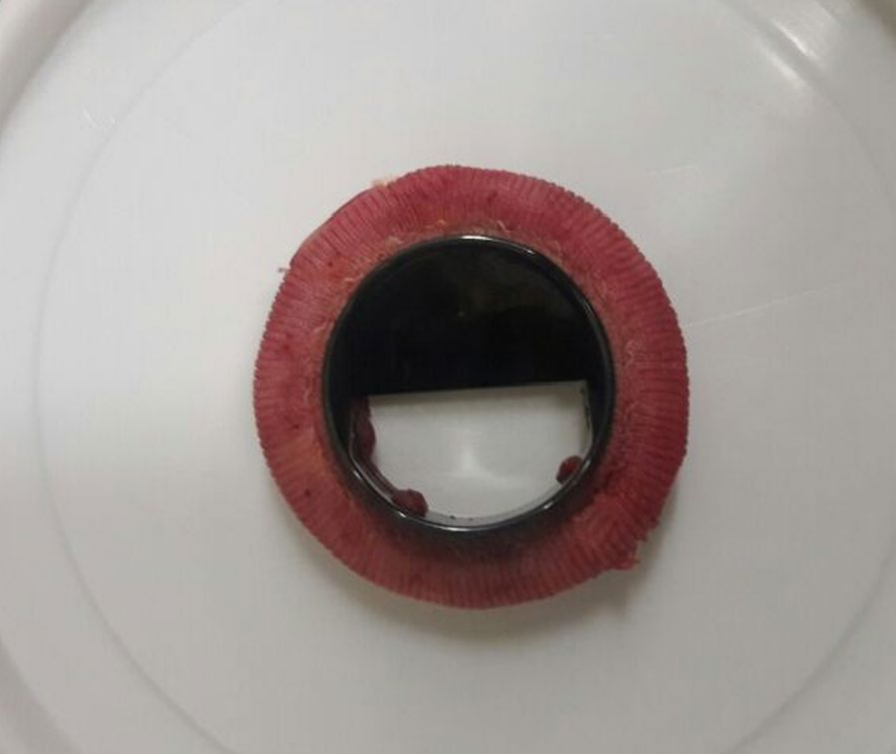
The explanted mechanical mitral valve prosthesis On-X with a single leaflet

A two-dimensional echocardiogram (2D echo) and fluoroscopy screening were done (Fig. 2). Both showed a single leaflet with normal mobility. The second leaflet was not visualized, and a working diagnosis of a stuck mitral valve prosthesis in a closed position was made. The patient underwent emergency redo mitral valve replacement. Intraoperatively on exploring the mitral valve prosthesis, a single leaflet was noted, and there was no pannus or clot formation (Fig. 3). The second leaflet was missing. The missing leaflet was not found inside the heart nor in the chest on an intraoperative transoesophageal echocardiogram. The valve was excised, and a size 25 mm On-X was implanted. We decided to reimplant the On-X valve because we could not find the literature on previous On-X valve leaflet fracture cases at the time of surgery. We assumed it was an isolated case of a possible manufacturing defect. Off bypass, all the patient’s peripheral pulses were present. He had no ischemic limb, organ or cerebrovascular accident /transient ischaemic attack at any stage. We elected to locate and remove the leaflet once he was out of the intensive care unit.

**Fig. 4:**
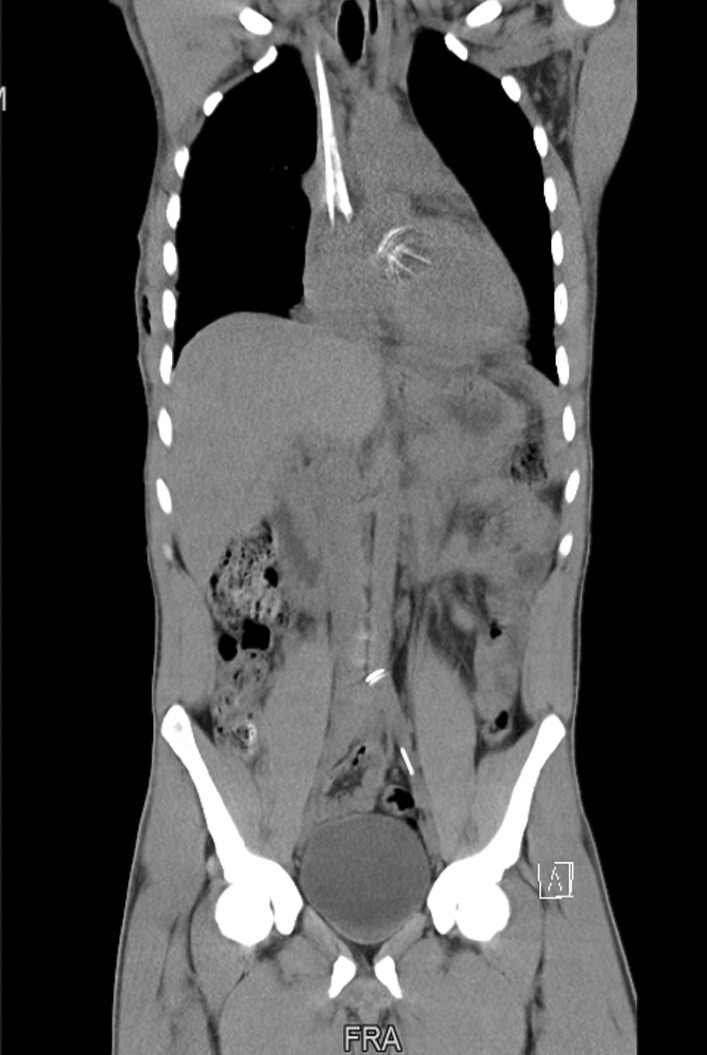
Computed tomography of the whole body showing the mechanical mitral valve leaflet at the abdominal aortic bifurcation and left external iliac artery

**Fig. 5:**
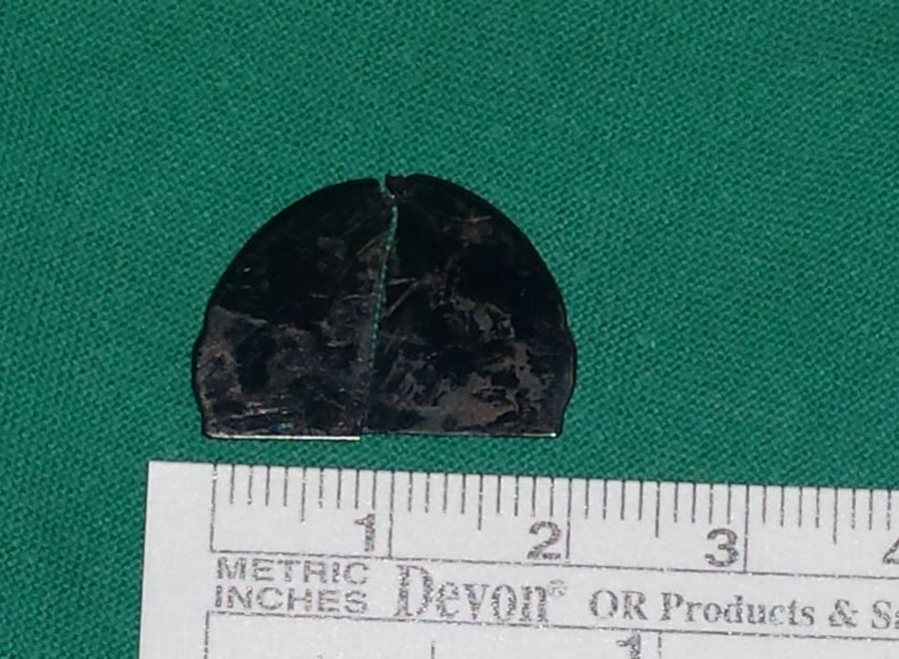
The fractured and embolized leaflet extracted from the aorta and external iliac artery

Post-operatively an unenhanced computer tomography (CT) of the whole body was done, which showed the leaflet had embolized to the aortic bifurcation and another fragment to the proximal left external iliac artery (Fig. 4). The patient underwent a successful open removal of the fractured leaflet via midline laparotomy on day 13 post-cardiac surgery (Fig. 5). His post-operative course was uneventful, and an echocardiogram showed normal functioning mitral valve prosthesis with a good ventricular function. He was discharged, and his follow-up at the outpatients department continues. The valve was sent to On-X for examination. The valve report stated that the mechanism of valve failure was noted to be cavitation. There was no definitive root cause found, and it was not possible to conclusively eliminate iatrogenic damage and patient factors, but no manufacturing defects were found.

## Discussion

Primary structural failure of mechanical valve prosthesis in the form of leaflet fracture and embolization with the modern mechanical valve prosthesis is infrequent [1–3]. The On-X valve is a new generation mechanical valve with a design supposedly optimized for better hemodynamic performance. There are only three cases reported in the literature of On-X valve leaflet escape, which makes it improbable that a leaflet escape could be an initial diagnosis. We suspect that the valve fractured first and then embolized as it was in 2 pieces. However, the report did not conclusively support this theory. Gobel et al. reported the first fatal case of leaflet escape with the On-X valve in the aortic position. Kayegama et al. and Amoros Riviera et al. reported two cases of On-X mechanical mitral valve leaflet fracture and embolization [3, 4, 6]. The site of embolization reported was the left iliac artery, abdominal aorta and aortoiliac bifurcation. Two patients had fatal outcomes in the three cases reported with On-X mechanical valve leaflet escape [3, 4, 6]. Amoros Riviera et al. observed that one of the hinges had ruptured in the explanted valve; however, the mechanism of failure in the other two reported cases is not reported.

In patients presenting in cardiogenic shock or with pulmonary oedema with severe regurgitation on a background history of a previous valve replacement, a mechanical valve failure in the form of thromboembolism or pannus formation is usually the cause [1–6]. However, we would like to highlight leaflet embolization as another possible cause and the need for prompt diagnosis and surgical treatment as this may be lethal.

Transthoracic or transoesophageal echocardiography can be used to diagnose a missing leaflet. Fluoroscopy screening of the valve is also an alternative. However, both transthoracic echocardiography and fluoroscopy could not clearly identify the missing leaflet. In cases where the embolized leaflet is not found inside the heart intraoperatively or fragmentation is suspected, a full-body CT is reliable for localizing escaped leaflets [2–4]. The common sites for leaflet embolization have been the abdominal aorta, and iliac arteries [1–6]. After the diagnosis, patients should undergo emergency surgery for valve replacement.

There is no consensus regarding removing the embolized leaflets if there is no threatened limb or ischemic complications. We decided to remove the fractured leaflets as we feared the risk of the fragments lacerating the aorta or eroding into the vessel wall. Catheter removal was considered but not undertaken due to the possible risk of the fractured ends of the leaflets lacerating the vessel. We removed the embolized leaflets surgically.

## Conclusions

Structural valve failure with leaflet fracture and embolization is an extremely rare complication and may be fatal. Patients with cardiogenic shock or severe valve incompetence with a previous history of a prosthetic cardiac valve should get an echocardiogram without delays. We could not identify a cause for this prosthetic failure conclusively, and there are very few cases reported in the literature. Still, clinicians should be aware that leaflet fracture and embolization with On-X valves are possible.

## Data Availability

The data is not publicly available but available from the author on request.

## Abbreviation

CT: Computer tomography
